# Perceived Health Benefits of Ketogenic Diets in Lebanon Are Tempered by Challenges With Sustainability and Restrictive Eating

**DOI:** 10.1002/fsn3.72474

**Published:** 2026-10-25

**Authors:** R. Rizk, H. Fakih, R. El Helou, L. Alaaedeen, M. L. Kayal, J. Jabbour

**Affiliations:** ^1^ Department of Nutrition and Food Science, School of Arts and Sciences Lebanese American University Byblos Lebanon; ^2^ Institut National de Santé Publique d'Epidémiologie Clinique, et de Toxicologie (INSPECT‐LB) Beirut Lebanon; ^3^ Division of Human Nutrition and Health, Global Nutrition Wageningen University and Research Wageningen the Netherlands

**Keywords:** dietary habits, ketogenic diets, lebanon, motivation

## Abstract

Ketogenic diets (KD) are increasingly being utilized for health promotion, yet little is known about their adoption and sustainability. This study explores the motivations, practices, and challenges of adults following KD in Lebanon. Adults residing in Lebanon and following KD were approached for this online cross‐sectional study through social media and dietitian networks. The survey assessed motivations, practices including restrained eating, and challenges associated with following KD. We recruited 202 participants (mean age: 35 years; 78% female; university education: 87%; average duration of following KD: 15 months). Weight loss was the main reason for starting KD (60%). Information about KD was sourced from social media (60%), dietitian (43%), websites/blogs (43%), and friends (25%). Only 9% consulted health professionals. Barriers for following KD included cost (54%), giving up favorite foods (48%), and limited food options (44%). Around two thirds (65%) consumed less than two cups of vegetables daily and 44% used predominantly olive oil for cooking. Reported average drop in body mass index was from 30 to 25.6 kg/m^2^. Many reported going off the diet (69%) and weight cycling (85% of previous KD followers). Despite reported improvements in body shape (85%), physical health (62%), and skin health (55%), restrained eating behaviors were common, and around half of the participants (49%) believed KD are sustainable. KD are followed in Lebanon primarily for weight loss. While participants perceived improved mental and physical well‐being, significant barriers and restrictive eating patterns were reported. Future longitudinal studies should measure health and nutritional outcomes.

## Graphical abstract

Among 202 adults in Lebanon following a ketogenic diet, weight loss was the main motivation and social media the leading information source. Although participants reported perceived improvements in body composition, physical health, and eating habits, cost, limited food choices, dietary restriction, and concerns about sustainability and weight regain were common challenges.

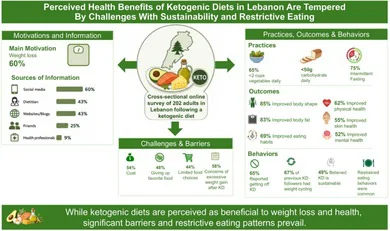

## 1 Introduction

Ketogenic diets (KD) are high‐fat, low‐carbohydrate dietary regimens (McGaugh and Barthel 2022). They are designed to induce a metabolic state of ketosis, where the body uses fat as its primary fuel source instead of carbohydrates. These diets involve a significant reduction in carbohydrate intake, typically to 5%–10% of total calories, while increasing fat consumption to about 70%–75%, with the remaining 20%–25% coming from protein (McGaugh and Barthel 2022). Several variations of KD exist: The standard KD, the most common regimen involving about 20 to 50 g of carbohydrate consumption per day; the targeted KD, which includes a small amount of fast‐digesting carbohydrates around workouts to provide extra energy for exercise; the cyclic KD, which alternates between periods of higher carbohydrate intake and strict ketogenic dieting; and the high‐protein KD, similar to standard KD with a higher proportion of protein reaching 35% of total calories (Barzegar et al. 2021).

Originally developed in the 1920s to treat epilepsy (Wheless 2008), KD were recently popularized for their other potential benefits through weight loss success stories, celebrity endorsements, and social media influence (Rana and Arora 2025). KD are suggested to improve metabolic abnormalities including weight loss, lipid profile, and glycemic control in individuals with Type 2 diabetes (Wong et al. 2021; Zhou et al. 2022), enhance cancer treatment (Tan‐Shalaby 2017), benefit various neurological and mental health disorders (Garner et al. 2024; Tillery et al. 2021), and boost endurance performance (Chamma et al. 2025; Chung 2023).

While KD offers numerous advantages, they have also been associated with possible adverse effects, including fiber and micronutrient deficiencies due to the restriction of carbohydrate‐rich foods (Andrewski et al. 2022). Moreover, some individuals may experience symptoms known as the “keto flu” during the initial adaptation phase, including headache, fatigue, dizziness, nausea, irritability, difficulty concentrating, and constipation (Batch et al. 2020). Additionally, KD may be associated with the development of kidney stones, hepatic steatosis, and hypoproteinemia in the long run (Batch et al. 2020). Mixed findings exist on their long‐term efficacy and safety (Batch et al. 2020; Masood et al. 2023; Popiolek‐Kalisz 2024).

Key factors appear to enhance the knowledge and perception of KD in the Arab region, including dietitian support, higher educational levels, lower body mass index (BMI), and middle age (Alhaj et al. 2021). However, perceptions regarding the challenges faced and ease of following the KD as a lifestyle are not conclusive (Alhaj et al. 2021; Shalabi et al. 2021). In light of this research gap, this study aims to assess the motivations, practices, and challenges of following KD among adults following this regimen in Lebanon while exploring restrained eating behaviors.

## 2 Methods

### 2.1 Study Design

This cross‐sectional study used an online questionnaire to investigate the practices of adults following KD in Lebanon, identify the motivations and challenges related to following these diets, and explore the extent of restrained eating in this study population. This study was reported in accordance with the Strengthening the Reporting of Observational Studies in Epidemiology (STROBE) guidelines (Data S1). Healthy individuals aged 18 years and above, following KD at the time of contact, residing in Lebanon, and able to read and write English or Arabic were included in the study. Participants were identified as following KD based on self‐reporting. There were no exclusion criteria applied.

Data collection spanned from February 2023 to August 2024. The questionnaire was administered via Google Forms and the link to the questionnaire was disseminated using social media platforms: Facebook, WhatsApp, Instagram, and LinkedIn through the personal accounts of the researchers, targeted groups, as well as personal contacts with key dietitians prescribing KD in Lebanon.

### 2.2 Data Collection Tool

The questionnaire was developed in English and translated to Arabic. Both versions were used for data collection. The questionnaire consisted of several sections including open‐ended, multiple choice, and rating questions:

1. Sociodemographic variables: This section assessed the participants' age, sex, educational level, and socioeconomic status.
2. Knowledge and Practices Related to KD: Participants were asked about the sources of information, prior knowledge, factors that help them stay on track, whether they consulted with a healthcare professional prior to following KD, as well as their weight changes when on KD. This section also included questions about participants' sources of information on the KD, dietary and supplement intake while following the diet, awareness of when their body enters ketosis, and history of KD adherence.
3. Motivations and challenges: this section explored obstacles participants encountered while following KD. Questions addressed issues such as difficulties in maintaining the diet, experiencing the keto flu symptoms, and the social and financial impacts of adhering to the diet. The motivations section delved into the factors driving participants to adopt KD. This included questions about the factors that encouraged them to initiate KD, the experienced health benefits, and comparisons of KD with other diets the participants have experienced. Participants were asked to rate their level of agreement with the statement, “I see myself continuing this diet,” using a 5‐point Likert scale (1 = Strongly Disagree, 5 = Strongly Agree).
4. Dutch restrained eating scale: This 10‐item questionnaire validated in the adult Lebanese population (Saade et al. 2019) was used to assess the frequency of dieting behaviors by using a 5‐point Likert scale (never, seldom, sometimes, often, and very often). The total score was obtained by dividing the total items score by the total number of items, whereby a higher score indicates higher restrained eating.
5. Physical activity: The physical activity level was assessed using the International Physical Activity Questionnaire—Short Form (IPAQ‐SF), a validated self‐report instrument that captures the frequency and duration of vigorous‐ and moderate‐intensity activities, walking, and sitting over the past 7 days. Responses were converted into metabolic equivalent (MET) minutes per week, allowing participants to be classified into low, moderate, or high physical activity levels based on established scoring criteria (Craig et al. 2003).

### 2.3 Ethical Considerations

The study received ethical approval from the institutional review board of the Lebanese American University (LAU) (LAU.SAS.RR3.26/Jan/2023). Participants consented to participate in the study by clicking on the “Accept” button on the first page of the online questionnaire. Participants had the choice to withdraw from the study at any time by clicking the “Decline” button, which directly closed the survey. Confidentiality of participants was protected where no identifiers were collected.

### 2.4 Sample Size Calculation

The sample size was calculated based on the anticipated difference in the mean Dutch Restrained Eating Scale score between KD followers and the adult Lebanese population (Saade et al. 2019). Assuming a small effect size of 0.2, an alpha of 0.05 and a power of 0.80, the calculation led to a minimum required sample of 199 participants. The sample size calculation was performed using G*Power software 3.1.9.7.

### 2.5 Statistical Analysis

Descriptive statistics were employed to summarize the sociodemographic characteristics, sources of information, dietary practices, and perceptions related to the KD. Categorical variables were expressed as frequencies and percentages, while continuous variables were presented as means and standard deviations (SD). To assess the difference between the Dutch Restrained Eating Scale mean score in our sample and that of a reference group (Saade et al. 2019), we conducted a one sample *t*‐test and then calculated Cohen's d. The mean and standard deviation for our sample came from our descriptive analysis, while the values for the comparison group were provided by the author, as they were not reported in the published article. We conducted exploratory univariable and multivariable regression analyses to better identify predictors of restrained eating using the ENTER method. The restrained eating score was included as the dependent variable, and independent variables were selected based on their relevance and previous literature. The variables with a *p* value of 0.2 or less in the univariable model were included in the multivariable model. Analysis was performed using IBM SPSS Statistics, version 28.

## 3 Results

A total of 202 participants were included in the study. With a mean age of 35 ± 11 years, the sample was predominantly female (78%) and university educated (87%). Almost half (48%) were classified as having a normal BMI at the time of data collection, while 37% were in the overweight category, and 15% had obesity. Participants were following KD for a mean period of 15 ± 28 months during which their reported mean BMI reduced from 30 ± 6.8 kg/m^2^ prior to following the diet to a current mean BMI of 25.6 ± 4.4 kg/m^2^. Physical activity data were available for a subset of participants (*n* = 69), due to incomplete responses to the IPAQ‐SF questionnaire. Around 41% of the 69 participants had a low physical activity level (Table 1).

**TABLE 1:** Sociodemographic characteristics of adults following ketogenic diets.

Variable		Overall (*n* = 202)
Age (years), mean (SD)		35 (11)
Female, *n* (%)		157 (78)
Educational level, *n* (%)	High school/equivalent or below	24 (12)
	University degree or equivalent	175 (87)
	Prefer not to respond	3 (1.5)
Monthly income (USD), *n* (%)	< 200	17 (8.4)
	200–999	62 (31)
	1000–2999	57 (28)
	≥ 3000	66 (33)
BMI current, Kg/m^2^, *n* (%)	Normal weight	97 (48)
	Overweight	75 (37)
	Obese	30 (15)
Crowding Index, *n* (%)	Less than 1	91 (45)
	1 and higher	111 (55)
Physical activity level, *n* (%)^a^	Low	28 (41)
	Moderate	26 (38)
	High	15 (22)
Duration following the KD (months), mean (SD)		15 (28)
BMI prior KD, Kg/m^2^, mean (SD)		30 (6.8)
BMI current (Kg/m^2^), mean (SD)		25.6 (4.4)

Abbreviations: BMI, body mass index; KD, ketogenic diets.

^a^ Physical activity level available for 69 participants.

Over half of the participants (59%) first heard about KD more than 3 years prior to the date of data collection, and half heard about it from friends, family, and/or coworkers. The primary sources of learning were social media (60%), dietitian (43%), and website/blog (43%). Participants reported that anticipating good results (50%) and experiencing satisfactory results (64%) are the most important factors that help them stay on track. Among the 48% who were part of a support group, 37% joined a Lebanese or Arabic support group, 8% an international group, and 40% joined both groups. Moreover, 79% reported that the support groups help them get new recipes, while 59% mentioned that the support group keeps them motivated to follow the diet (Table 2).

**TABLE 2:** Information and support sources of adults following ketogenic diets.

Survey question, *n* (%)		Overall, (*n* = 202)
When was the first time you heard of the KD?	< 1 year	21 (10)
	1 to less than 2 years	24 (12)
	2 to less than 3 years	38 (19)
	3 years and above	119 (59)
How did you first learn about the KD?	Friends/family/coworkers	100 (50)
	Gym/coach/trainer	10 (4.9)
	Health practitioner	18 (8.9)
	Dietitian	48 (24)
	Media	32 (16)
	Website/blog	54 (27)
	Social media networks	103 (51)
How do you obtain information on the KD?	Friends/family/coworkers	50 (25)
	Gym/coach/trainer	8 (3.9)
	Health Practitioner	18 (8.9)
	Dietitian	87 (43)
	Media	33 (16)
	Website/blog	86 (43)
	Mobile application	30 (15)
	Social media networks	121 (60)
What helps you stay on track with your diet?	Support group	33 (16)
	Supportive friends/family members	60 (30)
	The diet is easy to follow	68 (34)
	Anticipating good results	100 (50)
	Experiencing satisfactory results	129 (64)
Are you part of a support group?	Yes, Lebanese or Arabic groups	48 (37)
	Yes, international groups	10 (7.6)
	Yes, both groups	38 (40)
How does being part of a support group help you?	It keeps me motivated to follow the ketogenic diets	60 (59)
	It helps me get new recipes	82 (79)
	It gives me space to ask questions	53 (51)
	It does not help me much	2 (1.0)

Abbreviation: KD, ketogenic diets.

Approximately 69% of participants reported being able to identify when they are in ketosis, mainly based on symptoms (85%). Most participants (58%) consumed two meals daily, and 61% consumed a low amount of carbohydrate (less than 50 g daily). Perceived adherence to KD was self‐rated as good or very good by 65% of participants. Around 15% of participants reported daily consumption of fried foods, 65% consumed less than two cups of vegetables per day, 44% mainly used olive oil as a fat source for cooking, and 35% used animal ghee and butter. Around 75% reported practicing intermittent fasting while following KD. As for supplement intake, participants mainly consumed vitamin D (35%), magnesium (24%), multivitamins (10%), and fish oil (10%) (Table 3).

**TABLE 3:** Dietary practices of adults following ketogenic diets.

Survey question, *n* (%)		Overall, (*n* = 202)
Do you believe you can tell when your body is in ketosis?	No	37 (18)
	Yes	140 (69)
	I am not sure what Ketosis is	25 (12)
How can you tell when your body is in ketosis?	Based on symptoms that I feel	125 (85)
	I measure ketone levels	35 (23)
Do you measure/weigh/calculate your food intake?	Always	19 (9.4)
	Often	61 (30)
	Occasionally	46 (23)
	Rarely	35 (17)
	Never	41 (20)
On average, how many meals do you consume per day?	Less than one (alternate fasting)	6 (2.9)
	One	17 (8.4)
	Two	118 (58)
	Three or more	60 (30)
How many grams of carbs do you consume per day	< 25 g	77 (38)
	25–49 g	46 (23)
	50–89 g	20 (10)
	90 and above grams	11 (5.5)
	I am not sure	47 (23)
How do you describe your overall adherence to the KD?	Excellent	27 (13)
	Very good	65 (32)
	Good	67 (33)
	Fair	34 (17)
	Poor	7 (3.5)
	Very poor	2 (1)
Describe the frequency of your consumption of processed meats	Rarely/Less than once per month	103 (51)
	Monthly	41 (20)
	Weekly	44 (22)
	Daily	14 (7)
Describe the frequency of your consumption of fried foods	Rarely/Less than once per month	70 (35)
	Monthly	38 (19)
	Weekly	64 (32)
	Daily	30 (15)
Which of the ingredients below do you mainly use for cooking?	Animal ghee	15 (7.4)
	Butter	57 (28)
	Olive oil	89 (44)
	Vegetable oil	40 (20)
	Vegetable shortening	1 (0.5)
How would you describe your daily intake of vegetables?	Rarely	7 (3.5)
	< 1 cup per day	45 (22)
	1–2 cups per day	80 (40)
	> 2 cups per day	70 (35)
Do you practice intermittent fasting while following the KD?	Yes, 5:2 diet	12 (6)
	Yes, time‐restricted feeding	63 (31)
	Yes, variable	77 (38)
Do you take any of the following nutritional supplements?	Magnesium	49 (24)
	Potassium	6 (3)
	Sodium	1 (0.5)
	Vitamin D	71 (35)
	Multivitamin	21 (10)
	Fish oil	21 (10)
	Pre/probiotic	10 (5)
	Protein/Amino acids	3 (1.5)
	Exogenous ketones	0 (0)

Abbreviation: KD, Ketogenic diets.

Almost 69% of participants allowed themselves to get off KD. Among those, 26% did so for 2–3 days monthly, and 14% once per week. Participants who previously followed KD reported weight loss averaging 4.1 ± 2.4 kg per month. Of those participants, 85% experienced weight cycling: 15% maintained their weight loss, 55% regained some weight, 24% regained all the weight lost, and 6% gained more than starting weight (Table 4).

**TABLE 4:** Weight cycling characteristics after stopping ketogenic diets.

Variable		Overall (*n* = 202)
Number of days getting off KD, *n* (%)	Zero days	61 (30)
	1 day monthly	53 (26)
	2–3 days monthly	53 (26)
	1 day weekly	28 (14)
	2 days or more weekly	7 (3)
Number of previous KD, *n* (%)	None	117 (58)
	One	47 (23)
	Several times	38 (19)
		**Overall (*n* = 85)** ^a^
Weight lost (kg) per month if KD were previously followed, mean (SD)		4.1 (2.4)
Weight cycling after stopping KD, *n* (%)		72 (85)
Weight status after stopping KD, *n* (%)	Maintained lost weight	13 (15)
	Regained some weight	47 (55)
	Regained all the weight	20 (24)
	Gained more than starting weight	5 (6)

Abbreviations: BMI, body mass index; KD, ketogenic diets.

^a^ These characteristics were studied for the sample that had a previous attempt of following ketogenic diets.

The primary motivation for starting KD was weight loss (60%), followed by overall health improvement (11%). However, participants reported many challenges including the cost of the diet (54%), giving up favorite foods (48%), and limited food choices (44%). Around 73% reported that the diet was more expensive than their usual diet. Among other challenges, participants experienced keto flu symptoms including headaches (42%), constipation (34%), mood swings (31%), and low energy (31%). When asked about the impact KD had on their social life, 50% of participants claimed that it had limited their options when going out, and 14% reported feeling compelled to cancel some plans because of adopting KD (Table 5). Participants also highlighted the positive impacts of following KD, including improvements in body shape (85%), body fat (83%), eating habits (69%), physical health (62%), skin health (55%), mental health (52%), blood tests (43%), and need for medication (26%) (Figure 1). Almost 49% of participants either agreed or strongly agreed that KD is a sustainable way of eating, while more than half (53%) reported genuinely enjoying these diets. Additionally, 69% reported agreeing or strongly agreeing that they had observed lasting effects from KD that they consider important. Nevertheless, around 35% acknowledged difficulty in balancing daily responsibilities while following KD, 58% expressed concerns about gaining excessive weight after discontinuing the diets, and nearly 49% indicated significant worry about the risk of “falling off” the diets (Figure 2).

**TABLE 5:** Motivations and challenges of adults following ketogenic diets.

Survey question, *n* (%)		Overall (*n* = 202)
What was your primary reason for choosing to follow a KD?	Weight loss	122 (60)
	Hunger/satiety	9 (4.5)
	Energy/stamina	1 (0.5)
	Athletic performance	1 (0.5)
	Overall health	23 (11)
	Mental performance	6 (3)
	Inflammation/pain management	7 (3.5)
	Epilepsy	2 (1)
	Blood sugar control/diabetes	13 (6.4)
	Future disease prevention	9 (4.5)
	Food allergies/sensitivities	1 (0.5)
In your opinion, what are the obstacles for following a KD?	No problem	22 (11)
	Social acceptance (friends, family)	39 (19)
	Lack of time/motivation	15 (7.4)
	Limited food choices/boredom from the same dietary options	88 (43.6)
	Cost	109 (54)
	Giving up favourite foods	97 (48)
	Lack of cooking knowledge or skills/difficulty preparing meals	35 (17)
	Availability of food outside the home	62 (30.7)
What is the financial impact of this diet compared to your usual diet?	More expensive	147 (73)
	Less expensive	13 (6.4)
	Same as my usual diet	42 (21)
Did you experience any of the keto flu symptoms?	None	49 (24)
	Brain fog	32 (16)
	Dizziness	54 (27)
	Headaches	85 (42)
	Constipation	69 (34)
	Nausea	37 (18)
	Low energy	62 (31)
	Mood swings	62 (31)
How has the KD impacted your social life?	Did not affect my social life	92 (46)
	Limited my options while going out	100 (50)
	Motivated me to cancel some plans	28 (14)

Abbreviation: KD, ketogenic diets.

**FIGURE 1:**
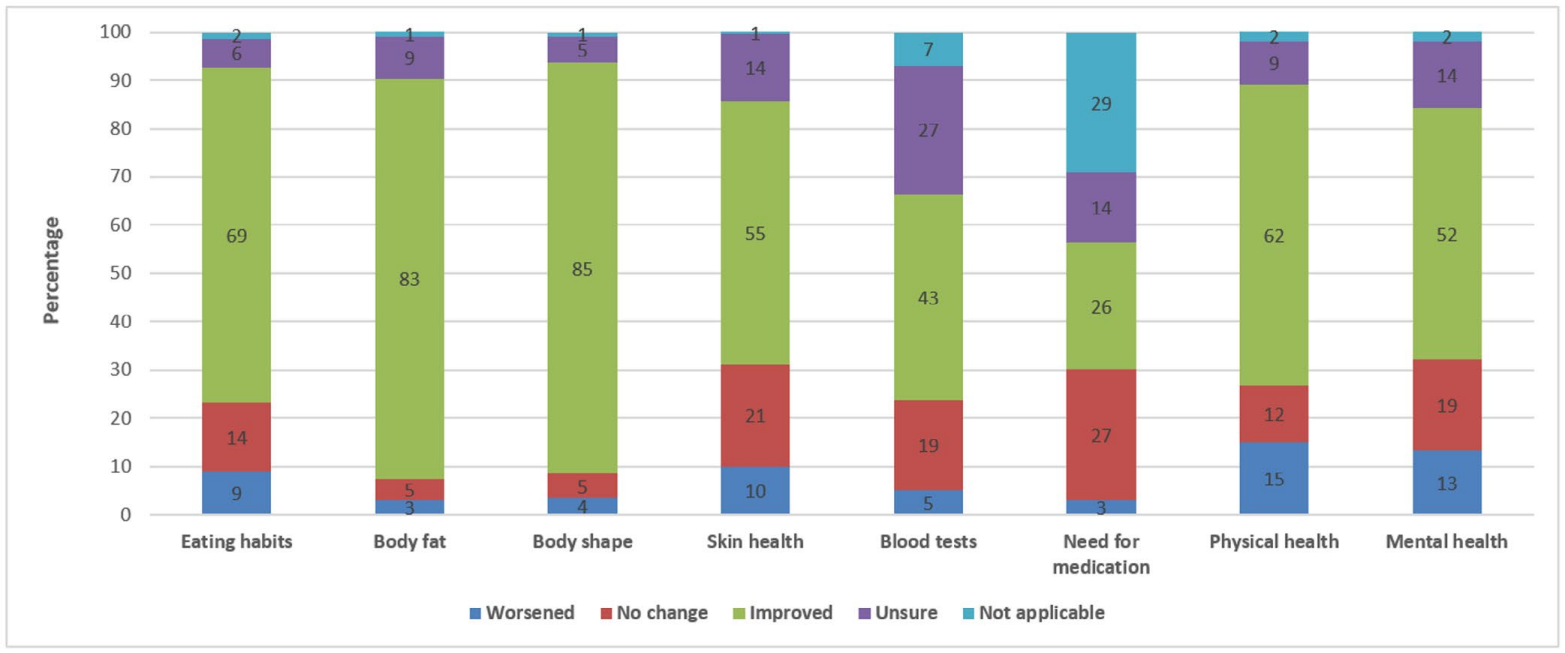
Self‐reported changes associated with following ketogenic diets. This stacked bar chart illustrates the reported changes in various health and wellness metrics for adults following ketogenic diets. Data for eight distinct categories (eating habits, body fat, body shape, skin health, blood tests, need for medication, physical health, and mental health) are presented. Each bar represents a single health category and is segmented to show the percentage of participants who reported their condition had worsened (dark blue), showed no change (red), improved (green), were unsure (purple), or found the category not applicable (light blue). The data highlight a general trend of improvement in physical‐related metrics while also indicating no change noted in mental health and the need for medication.

**FIGURE 2:**
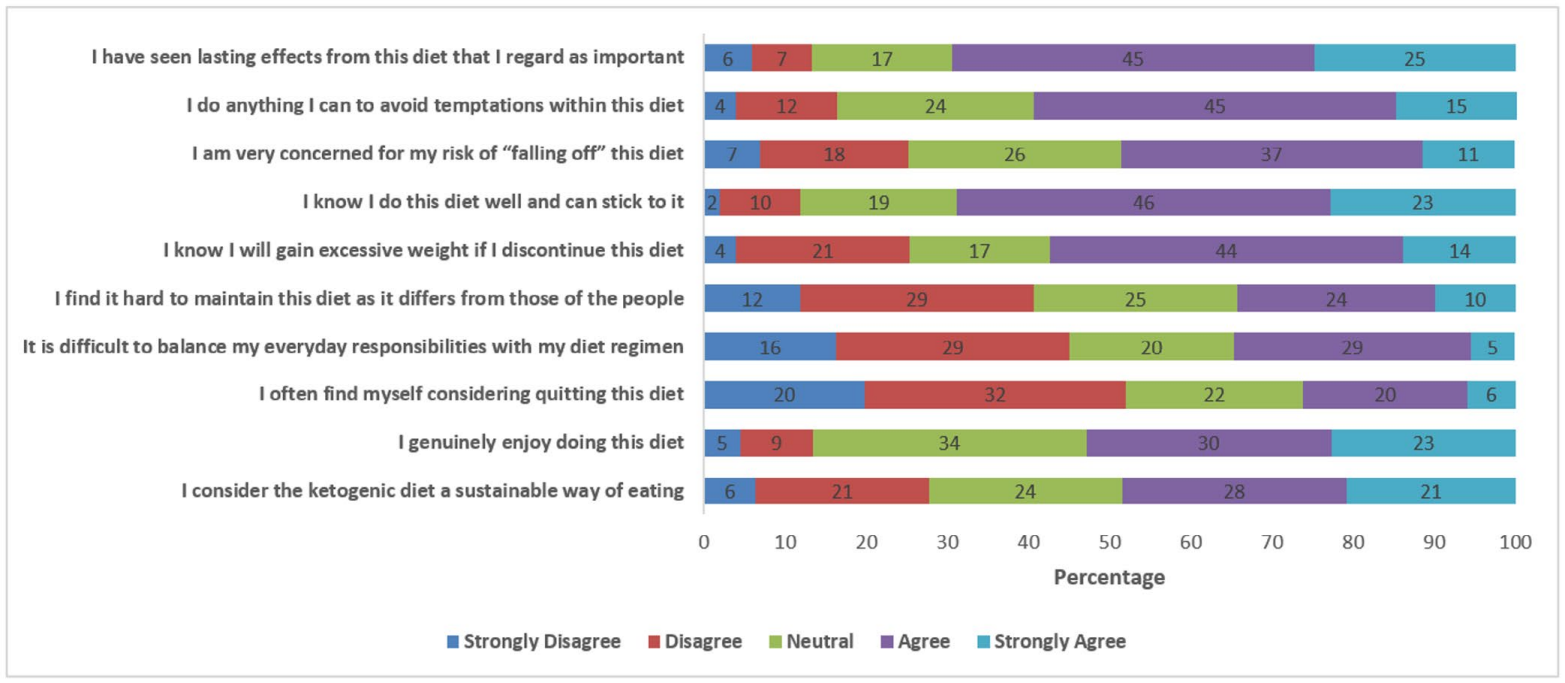
Reported attitudes and challenges in following ketogenic diets. This stacked bar chart presents the self‐reported perceptions and attitudes of adults following a ketogenic diets. The data are organized into 10 distinct statements related to diet sustainability, adherence, social challenges, and perceived effectiveness. Each horizontal bar is segmented to illustrate the percentage of respondents who selected one of five options on a Likert scale, ranging from Strongly disagree (dark blue) to Strongly agree (light blue). The figure highlights the perceived effectiveness and high commitment to the diet among participants, while also revealing significant challenges related to social integration, balancing daily responsibilities, and concerns about long‐term adherence and potential weight regain.

The mean score for the Dutch Restrained Eating scale in our sample was 3.18 ± 0.78, significantly higher than the mean of 2.55 ± 0.97 reported for the Lebanese adult population by Saade et al. (*p* < 0.001) (Saade et al. 2019). A moderate to large effect size was observed between the two reported means (Cohen's d = 0.67). Participants' responses to the Dutch Restrained Eating Scale indicated that the majority exhibited restrained eating patterns while following KD. These behaviors included avoiding evening meals to prevent weight gain (49%), refraining from eating in‐between meals (50%), measuring food weight and watching exactly what they eat (58%), and reducing food intake after gaining weight (41%) (Figure 3).

**FIGURE 3:**
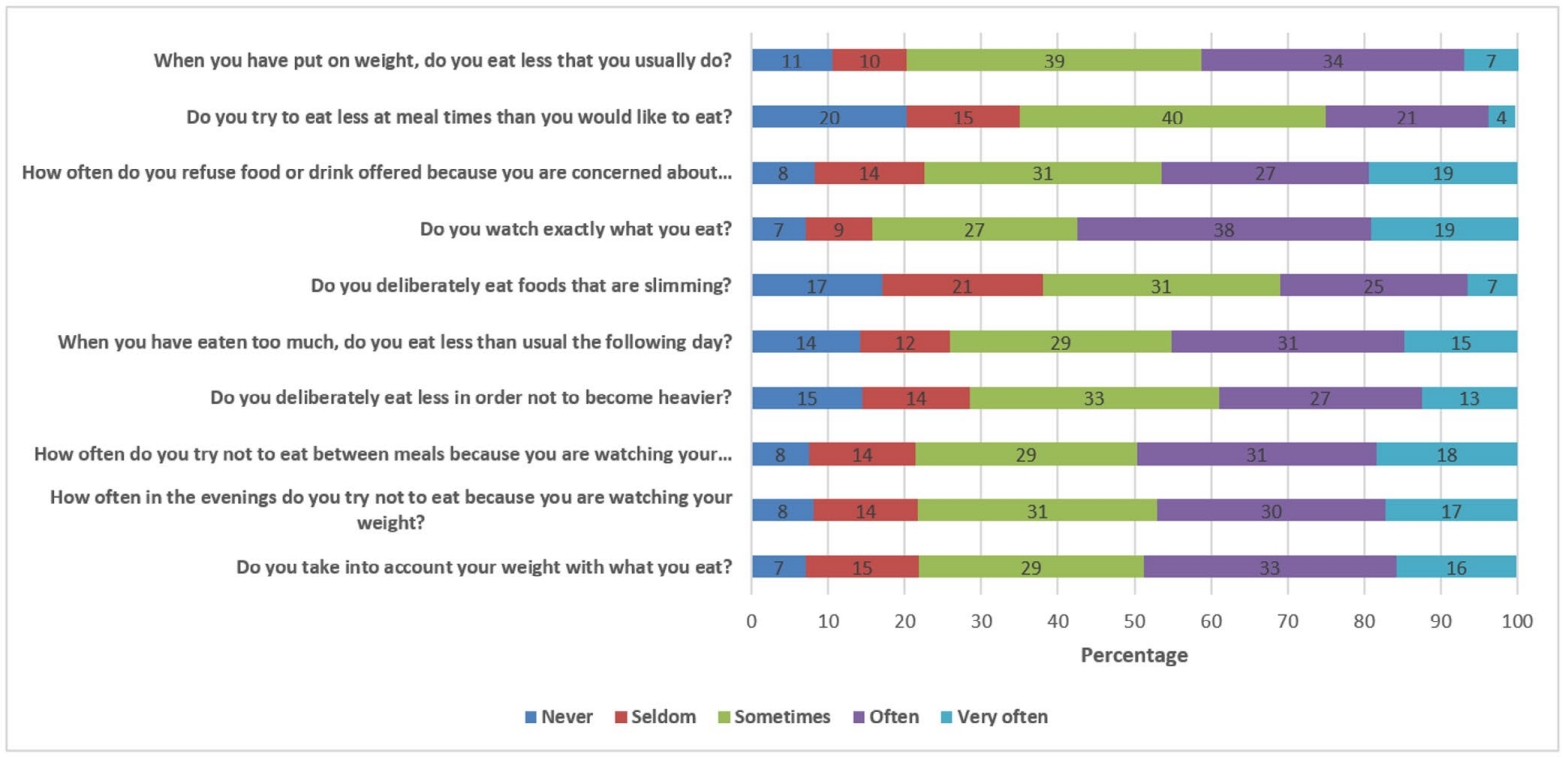
Measures of restrained eating from the Dutch restrained eating scale. This stacked bar chart illustrates the self‐reported frequency of restrained eating behaviors among adults following ketogenic diets. Each horizontal bar corresponds to a distinct question probing a dietary control or compensatory behavior. The data are presented on a frequency scale, with each segment of the bar representing the percentage of respondents who chose Never (dark blue), Seldom (red), Sometimes (green), Often (purple), or Very often (light blue). The figure demonstrates a widespread pattern of active and frequent dietary control, with a significant majority of participants reporting that they often or very often engage in behaviors such as monitoring food intake, skipping meals, or eating less to manage their weight.

In the regression analysis, three variables in the univariable model met the *p* ≤ 0.2 criterion for inclusion in the multivariable model (Table 6). Those are age (*p* = 0.065), duration of following KD (*p* = 0.118), and weight cycling (*p* = 0.169). Age was borderline significant in the multivariable model (*p* = 0.047). None of the remaining variables showed statistical significance at the multivariable level (Table 6).

**TABLE 6:** Univariable and multivariable linear regression of the predictors of the restrained eating score.

Variable	Univariable Regression				Multivariable Regression			
	Unadjusted Beta	SE	*p*	95% CI	Adjusted Beta	SE	*p*	95% CI
Age, years	−0.012	0.006	0.065	−0.02–0.001	−0.016	0.008	0.047	−0.03‐0
Gender	0.096	0.160	0.547	−0.22–0.412	—	—	—	—
BMI, Kg/m^2^	−0.029	0.093	0.760	−0.21–0.156	—	—	—	—
BMI prior to following the ketogenic diets, Kg/m^2^	0.064	0.083	0.442	−0.1–0.229	—	—	—	—
Duration of following the ketogenic diets (months)	−0.006	0.004	0.118	−0.01–0.001	−0.001	0.004	0.756	−0.01–0.01
Consultation of a healthcare professional	−0.061	0.132	0.642	−0.32–0.199	—	—	—	—
Joining a support group	0.110	0.131	0.403	−0.15–0.37	—	—	—	—
Educational level	−0.277	0.296	0.351	−0.87–0.312	—	—	—	—
Monthly household income	−0.039	0.068	0.574	−0.17–0.097	—	—	—	—
Physical activity level	0.026	0.058	0.648	−0.09–0.141	—	—	—	—
Weight cycling	0.269	0.195	0.169	−0.12–0.655	0.267	0.194	0.173	−0.12–0.65

*Note:* Dependent variable is the score of the Dutch Restrained Eating Scale. Gender: 1 = male, 2 = female; BMI: 1 = normal weight, 2 = overweight, 3 = obesity; consultation of a healthcare professional: 0 = no, 1 = yes; Joining a support group: 0 = no, 1 = yes; Educational level: 1 = High school or equivalent and below, 2 = university degree or equivalent; Monthly household income (USD): 1 = < 200, 2 = 200–999, 3 = 1000–2999, 4 = ≥ 3000; Weight cycling: 1 = maintained weight, 2 = regained weight after stopping the ketogenic diets.

Abbreviations: BMI, body mass index; CI, confidence interval; SE, standard error.

## 4 Discussion

We explored the motivations, practices, and challenges of adults in Lebanon following KD. Weight loss was the predominant motivation for adopting KD, with social media and friends/family being the main sources of information. A significant proportion of participants reported enjoying KD and experiencing positive effects on body composition, eating habits, and mental health. However, challenges such as financial cost, limited food variety, and social constraints were commonly reported. Notably, concerns about regaining weight after discontinuation and the difficulty of maintaining adherence were prevalent among participants. Features of restrained eating were evident in the sample; however, none of the variables in the regression analysis predicted restrained eating behaviors.

Aligning with findings from previous research, females (Alhaj et al. 2021; Garner et al. 2024) and young adults (Shalabi et al. 2021) constituted the majority of our sample. This highlights a potential sex and age‐related trend in KD adoption, raising the question of whether younger female adults are more likely to adopt KD or are overrepresented in online studies. Another key result was the participants' reliance on social media for information on KD, with minimal consultation of healthcare professionals, mirroring results of other studies in the Arab region (Alhaj et al. 2021; Shalabi et al. 2021). This finding also aligns with a previous study conducted among adults in Ghana where 92% of participants rely on online resources when seeking nutrition guidance (Quaidoo et al. 2018). The high reliance on social media as a source of information on KD highlights the growing influence of digital platforms on dietary decision‐making. While social media may improve accessibility to dietary information, misinformation and non‐evidence‐based recommendations remain significant concerns (Rana and Arora 2025). Additionally, our findings reveal lower rates of dietitians consultation compared with those reported by Alhaj et al., suggesting varied professional involvement across different settings (Alhaj et al. 2021). Dietitians' consultations seem to be lower in Lebanon and the Arab region compared with Western countries, such as the United Kingdom (Garner et al. 2024), highlighting a potential gap in accessibility to and/or affordability of credible nutritional care (Alameddine et al. 2013). This result was predictable in our sample as the majority of our participants rely on social media and friends for nutrition information. This reliance limits exposure to evidence‐based dietary counseling, whereas the KD requires planning to ensure nutritional adequacy. Without professional guidance, the risk of adopting overly restrictive dietary patterns increases, which reduces overall diet sustainability.

Long‐term adherence to KD is problematic, with dropout rates increasing beyond 6 months due to the restrictive nature of the diet (Li et al. 2023; Ross et al. 2021). In our study, a significant proportion of participants engaged in short‐term keto cycling, usually going off the diet for 3 days or less monthly, similar to what was reported by Alhaj et al. (2021). More than one half of our participants expressed fear of weight regain after stopping the diet, aligning with studies among humans and animals demonstrating a tendency for weight regain following weight cessation (Basolo et al. 2022; Paoli et al. 2015; Weber et al. 2022). While engaging in periodic breaks from the diet was common among our participants, almost half of them viewed KD as a sustainable way of eating.

Financial cost and social difficulties were significant barriers to adherence. Shalabi et al. (2021) reported similar findings, emphasizing that the cost of high‐quality fats and protein sources limits adherence in middle‐income countries. This concern was especially relevant at the time of study conduct given that the study was done during a severe economic crisis in Lebanon (Hausmann et al. 2023).

Other concerns associated with KD relate to the nutritional adequacy of the regimens. Given the reliance on fats and protein, deficiencies in fiber, vitamins, and minerals are common, especially when diets are self‐directed (Weber et al. 2022). The relatively low vegetable intake reported by participants may be due to the fact that KD limits the consumption of certain vegetables due to carbohydrate restrictions. This contributes to less consumption of fiber‐rich foods, potentially leading to gastrointestinal problems such as constipation (Tuck and Staudacher 2019).

Restrictive eating behaviors were notable findings in our study. The mean score for the Dutch Restrained Eating Scale was higher than that reported among the Lebanese adult population (Saade et al. 2019). Main traits observed included being very cautious about what to eat and reducing food portions for fear of weight gain. These trends of disordered eating align with concerns raised in previous research (Weber et al. 2022), but contradict the body of evidence showing the potential of the diet to treat disordered eating (Carmen et al. 2020; Scolnick et al. 2020). The restrictive nature of the diet posed a challenge in social settings especially given that Lebanese cuisine is traditionally rich in carbohydrates. This can make it difficult for individuals following KD to participate in shared meals, a main concern considering the crucial role of food culture in Lebanon (Hwalla and El Khoury 2008). Given the growing popularity of KD, particularly among young adults, future research should adopt a longitudinal approach to assess its long‐term effects on both physical and psychological well‐being.

## 5 Strengths and Limitations

This is the first study in Lebanon to explore KD in the country. It benefits from a diverse sample of individuals actively following KD, enhancing the relevance of its findings. By offering the questionnaire in both English and Arabic, the study ensured accessibility for participants with different language preferences. The questionnaire assessed various aspects of dietary behavior, including dietary practices, motivation, and restrained eating. Also, the use of a validated tool, which is the Dutch Restrained Eating Scale, adds credibility to the assessment of dieting behaviors.

Certain limitations must be acknowledged. The reliance on self‐reported data from an online questionnaire introduces the possibility of response bias, including social desirability bias, as well as recall bias, whereby participants may overestimate or underestimate their dietary behaviors. In addition, the study was prone to misclassification bias since following KD was based on self‐identification. Participants were asked to report carbohydrate intake, and some participants exceeded typical thresholds or were uncertain about their intake, reflecting variability in adherence. Moreover, non‐random sampling methods, such as recruitment through targeted social media groups and dietitian referrals, limit the generalizability of the findings and may have contributed to selection bias by overrepresenting individuals who are more health‐conscious or engaged in KD communities. Another limitation of this study is the use of a non‐validated questionnaire to assess participants' motivations, practices, and challenges of following KD due to the absence of a validated tool. This factor may impact the validity, reliability, and comparability of the findings. The cross‐sectional study design, along with the absence of a control group, limits the ability to establish temporal relationships, assess long‐term adherence to KD, and evaluate their long‐term health impacts. Lastly, the multivariable regression model was constrained by missing data on weight cycling, which was only assessed among participants with a prior history of discontinuing KD. This reduced analytic sample may have limited power to detect true associations, particularly for weight cycling itself.

## 6 Conclusion

This study provides key insights into the self‐reported motivations, practices, and challenges of adult followers of KD in Lebanon. It adds to the scarce literature exploring KD experiences in adults. Findings highlight the limited professional support, particularly in addressing nutritional adequacy and long‐term sustainability. While participants perceived improved mental and physical well‐being, significant barriers such as cost, social factors, and restrictive eating patterns were reported. Future research should measure longitudinal nutritional and health outcomes including nutritional adequacy, disordered eating, and cardiometabolic profile and compare them with other diets.

## Author Contributions

**R. Rizk:** conceptualization, visualization, data curation, writing – review and editing, supervision, formal analysis. **H. Fakih:** data curation, investigation, writing – review and editing. **R. El Helou:** data curation, writing – review and editing, formal analysis. **L. Alaaedeen:** data curation, investigation, writing – review and editing. **M. L. Kayal:** data curation, investigation, writing – review and editing. **J. Jabbour:** conceptualization, data curation, supervision, visualization, methodology, writing – review and editing, formal analysis.

## Funding

The authors have nothing to report.

## Conflicts of Interest

The authors declare no conflicts of interest.

## Supporting information


**Data S1:** STROBE statement—checklist of items that should be included in reports of cross‐sectional studies.

## Data Availability

The data that support the findings of this study are available on request from the corresponding author. The data are not publicly available due to privacy or ethical restrictions.
